# Supplementary material for: Altered Development of Gut Microbiota and Gastrointestinal Inflammation in Children with Post-Operative Hirschsprung’s Disease
Source: Int J Mol Sci. 2025 Oct 30;26(21):10570. doi: 10.3390/ijms262110570 (PMC12609776; doi:10.3390/ijms262110570)
Supplement: Supplementary file 1 [file ijms-26-10570-s001.zip › ijms-3846410-supplementary.pdf]

Supplementary Materials

Table S1. Average relative abundance of most abundant bacterial phyla in stool. Values are presented as percentages, highlighting that *Bacteroides* and *Firmicutes* are the most abundant phyla across the population.

Phylum	Proportion (%)
Bacteroidetes	41.45
Firmicutes	39.49
Actinobacteria	10.75
Proteobacteria	5.518
Verrucomicrobia	2.06
Fusobacteria	0.29
Cyanobacteria	0.28
Tenericutes	0.15

Table S2. Average relative abundance of most abundant bacterial genera in stool. Values are presented as percentages, highlighting that genera from *Bacteroides, Rhodococcus* and *Faecalibacterium* are the most abundant genera across the population.

Genus	Proportion (%)
Bacteroides sp	37.58
Rhodococcus	17.20
Faecalibacterium	11.03
Bifidobacterium	10.31
Alistipes	7.62
Prevotella 9	6.04
Pseudobutyrivibrio	5.24
Lachnospiraceae NK4A136 group	4.97

Table S3. Comparison of dietary intake variables between HD and HC. There was no difference between inf dietary intake of all dietary variables between individuals with HD and HC as demonstrated by p value > 0.05. Adjusted p values are also presented to account for multiple testing.

Variable	p value	Adjusted p value
kJwithDF	0.17453	0.425994
kJwithoutDF	0.171804	0.425994
Moisture	0.093446	0.425994
Protein	0.177627	0.425994
Fat	0.22413	0.425994

CHO_with_EToH	0.151042	0.425994
CHO_without_EToH	0.150967	0.425994
Starch	0.210531	0.425994
Sugar	0.112866	0.425994
Addedsugarsg	0.180466	0.425994
Freesugarsg	0.152961	0.425994
Fibre	0.524044	0.58337
Ash	0.185974	0.425994
Retinol	0.150164	0.425994
Beta_Carotene	0.51008	0.579257
ProvitA_Eq	0.503246	0.579257
Retinol_Eq	0.901229	0.901229
Thiamin	0.494582	0.579257
Riboflavin	0.198898	0.425994
Niacin	0.396567	0.497819
Niacin_Eq	0.256026	0.425994
Folate	0.156116	0.425994
Folic_Acid	0.709859	0.761485
Total_Folates	0.361952	0.478815
Diet_Folate_Eq	0.439533	0.540259
Vit_B6	0.053872	0.425994
Vit_B12	0.145071	0.425994
Vit_C	0.792842	0.820661
Alpha_tocoph	0.22763	0.425994
Vit_E	0.238703	0.425994
Calcium	0.148338	0.425994
Iodine	0.208869	0.425994
Iron	0.237763	0.425994
Magnesium	0.136628	0.425994
Phosphorus	0.15788	0.425994
Potassium	0.143189	0.425994
Selenium	0.365198	0.478815
Sodium	0.381358	0.489133
Zinc	0.246222	0.425994
Caffeine	0.257356	0.425994
Cholesterol	0.204797	0.425994
Trytophan	0.170293	0.425994
Sat_fat	0.169424	0.425994
Mono_fat	0.295263	0.450805
Poly_fat	0.350014	0.478815
Linoleic	0.319353	0.459557
Alpha_linolenic	0.744941	0.784848
epa	0.107314	0.425994
dpa	0.259928	0.425994
dha	0.098962	0.425994
Omega3	0.10637	0.425994

Trans_fat	0.30563	0.450805
Wholegrains	0.510532	0.579257
Resist_starch	0.291479	0.450805
Total_fibre	0.301068	0.450805
Insol_fibre	0.330457	0.464213
Soluble_fibre	0.247815	0.425994
